# Sequential Congruency Effects of Reverse Stroop Interference on Event-Related Potential Components for Go- and Nogo-Stimuli

**DOI:** 10.3389/fpsyg.2021.678647

**Published:** 2021-07-29

**Authors:** Kota Suzuki

**Affiliations:** Faculty of Education, Shitennoji University, Habikino, Japan

**Keywords:** reverse Stroop interference, Go/Nogo task, N1, N2, P3, cognitive control

## Abstract

Sequential congruency effects are observed in interference tasks, in which reaction times (RTs) are shorter for congruent stimuli preceded by congruent (cC) than incongruent stimuli (iC), and RTs are longer for incongruent stimuli preceded by congruent (cI) than incongruent stimuli (iI). These effects are interpreted as resulting from incongruent stimuli triggering attentional control in the next trial, which reduces cognitive control. This study aimed to examine sequential congruency effects on event-related potential (ERP) components for Go- and Nogo-stimuli. We used the hybrid reverse Stroop Go/Nogo task. The stimuli were Kanji characters, “赤” (i.e., red) and “青” (i.e., blue) painted in congruent and incongruent colors. Participants responded to one of the two characters (i.e, the Go-stimulus) and stopped responding to the other character (i.e., the Nogo-stimulus). The results indicated that the Nogo-N1 was reduced by trials preceded by incongruent stimuli compared with congruent ones, suggesting that color processing was inhibited by attentional control; however, there was no reduction in the Go-N1. In addition, the Nogo-N2 amplitudes were larger for cI than iI and iC than cC. On the other hand, the Go-N2 was not modulated by sequential modulation effects, which was lower for incongruent stimuli than congruent stimuli. These results indicate that the Nogo-N2 is involved in cognitive control, whereas the Go-N2 is associated with selection processing. These findings suggest that the modulation of sequential congruency effects of N1 and N2 required the response inhibition task demand; however, Go-P3 and Nogo-P3 amplitudes were the largest for cI. Therefore, the time range of ERP components might be related to the susceptibility of an interaction effect between response inhibition task demand and sequential congruency effects.

## Introduction

The participants conducting a Go/Nogo task are required to respond to one type of stimuli (i.e., Go-stimuli) and withhold responding to another type (i.e., Nogo stimuli). In addition, Go- and Nogo-stimuli are known to elicit different event-related potential (ERP) components. The distribution of the positive ERP component peaking from 300 to 500 ms, i.e., the P3, has been demonstrated at the parieto-occipital scalp sites for Go-stimuli (the Go-P3 or the P3b; Polich, 2012). The P3 has been shown at more frontal sites for Nogo-stimuli (the Nogo-P3 or the P3a; Fallgatter and Strik, 1999; Polich, 2012). It is well known that rare target stimuli enhance the Go-P3 than frequent stimuli in the oddball task (Polich, 2007, 2012). The Go-P3 amplitude is reduced as a function of target probability in the prior sequence (Duncan-Johnson and Donchin, 1977). It has been reported that the Go-P3 is associated with the performance of recall tasks (Karis et al., 1984). Therefore, the Go-P3 might be related to context updating operations and subsequent memory storage (Polich, 2007, 2012). On the other hand, the Nogo-P3 is enhanced by cues inducing a high degree of response preparation (Bruin et al., 2001; Smith et al., 2007; Randall and Smith, 2011). Participants with short reaction times (RTs) might require more response inhibition effort than those with long RTs. The Nogo-P3 was larger in participants with short RTs compared with long RTs (Smith et al., 2006), suggesting that the Nogo-P3 is related to response inhibition (Gajewski and Falkenstein, 2013; Huster et al., 2013; Suzuki et al., 2020).

The front-central negative ERP component peaking from 200 to 350 ms, i.e., N2, is also involved with the Go/Nogo task. The N2 amplitudes are larger for Nogo-stimuli (i.e., the Nogo-N2) than Go-stimuli (i.e., the Go-N2; Pfefferbaum et al., 1985); however, previous studies have indicated that the difference between the Nogo-N2 and the Go-N2 is not directly associated with differences in response inhibition efforts. There was no difference in the Nogo-N2 between participants with short and long RTs (Smith et al., 2006). Donkers and Van Boxtel (2004) administered the Go/Nogo task and the Go/GO tasks, in which responses with maximal force were required for GO-stimuli. That is, GO-stimuli did not require response inhibition. The results indicated that the Nogo-N2 and the GO-N2 were enhanced in the low probability condition compared with the equal probability condition (Donkers and Van Boxtel, 2004), showing that N2 was modulated independently of response inhibition.

The Nogo-N2 and the Go-N2 might be associated with cognitive control preceding motor responses (Gajewski and Falkenstein, 2013; Huster et al., 2013; Suzuki et al., 2020). Previous studies have examined the association between N2 and cognitive control during conflicts. Conflicts are assumed to occur when incongruent response representations are simultaneously activated (Botvinick et al., 2001; Yeung et al., 2004). A conflict in the Go/Nogo task is reflected by an incongruency between the representation for response execution and the representation for stopping the response (Stahl and Gibbons, 2007; Randall and Smith, 2011). The degree of conflict can be manipulated by the probability and the cue in the Go/Nogo task. Previous studies have demonstrated that the N2 could be modulated by the degree of conflict (Donkers and Van Boxtel, 2004; Smith et al., 2010; Randall and Smith, 2011). For example, representations for response execution and response termination are activated by Go- stimuli after a cue informing a Nogo-stimulus. A high degree of conflict might occur without any response inhibition demands of Go-stimuli after a cue informing a Nogo-stimulus because no response inhibition is required for Go-stimuli. N2 amplitudes for Go-stimuli were larger after a cue informing a Nogo-stimulus than a cue informing a Go-stimulus (Randall and Smith, 2011). These findings indicate that a more intense conflict requires more effort cognitive control, which enhances N2.

Conflicts in interference tasks are also well-studied, including the flanker task, the Stroop task, and the reverse Stroop task (Botvinick et al., 2001; Yeung et al., 2004). The stimuli of Stroop and the reverse Stroop tasks are words expressing a color painted in a congruent or an incongruent color. The participants in the Stroop task (Stroop, 1935) are asked to respond to the color, whereas they are asked to respond to the meaning of the word in the reverse Stroop task (Flowers, 1975). Typically, longer RTs are observed for incongruent than congruent stimuli, suggesting that the incongruency between the color and the meaning of the word causes a conflict. In addition, sequential congruency effects are observed in the behavioral results of the interference task; RTs are shorter for congruent stimuli preceded by congruent ones (cC) than incongruent ones (iC), and RTs are longer for incongruent stimuli preceded by congruent ones (cI) than incongruent ones (iI) (Gratton et al., 1992). The sequential congruency effects are interpreted as incongruent stimuli triggering the inhibition of irrelevant processing through attentional control in the next trial, which reduces the degree of conflict and effort for cognitive control (Botvinick et al., 2001; Kerns et al., 2004).

The sequential congruency effects on the N2 and the P3 have been reported in previous interference task studies (Jiang et al., 2013; Panadero et al., 2015; Larson et al., 2016). The N2 and the P3 were enhanced by cI than iI in the flanker task (Larson et al., 2016). The probability of incongruent stimuli reportedly modulated the N2 and the P3 (Jiang et al., 2013; Panadero et al., 2015). These findings suggested that the N2 was associated with control during conflicts, consistent with previous Go/Nogo task studies. The P3 was also involved with interference task conflicts, unlike the Go/Nogo task; however, this finding might indicate that a high degree of conflict requires a large response inhibition effort.

Interference tasks used in previous studies required a choice response (Jiang et al., 2013; Panadero et al., 2015; Larson et al., 2016). Previous studies have shown that Go- and Nogo-stimuli differently influence ERP components (Pfefferbaum et al., 1985; Polich, 2007, 2012). Hypothetically, Nogo-stimuli require a higher effort for cognitive control and response inhibition, whereas Go stimuli require a lower effort. Hence, it was assumed that ERP components for Go-stimuli are influenced only by the conflict degree, whereas ERP components for Nogo-stimuli are influenced by the synergistic effect of conflict degree and other factors, such as cognitive control and response inhibition. Therefore, the sequential congruency effects of interference tasks might be different between ERP components of Go- and Nogo-stimuli. A previous study (Groom and Cragg, 2015) examined the effects of flanker interference on ERP components for Go-stimuli, Nogo-stimuli, and choice response (i.e., a left- or right-hand response) stimuli and indicated that the N2 and the P3 were enhanced by incongruent relative to congruent stimuli (Groom and Cragg, 2015); however, little is known about the interference effects and sequential congruency effects of other tasks on ERP components for Go- and Nogo-stimuli.

**Figure 1:**
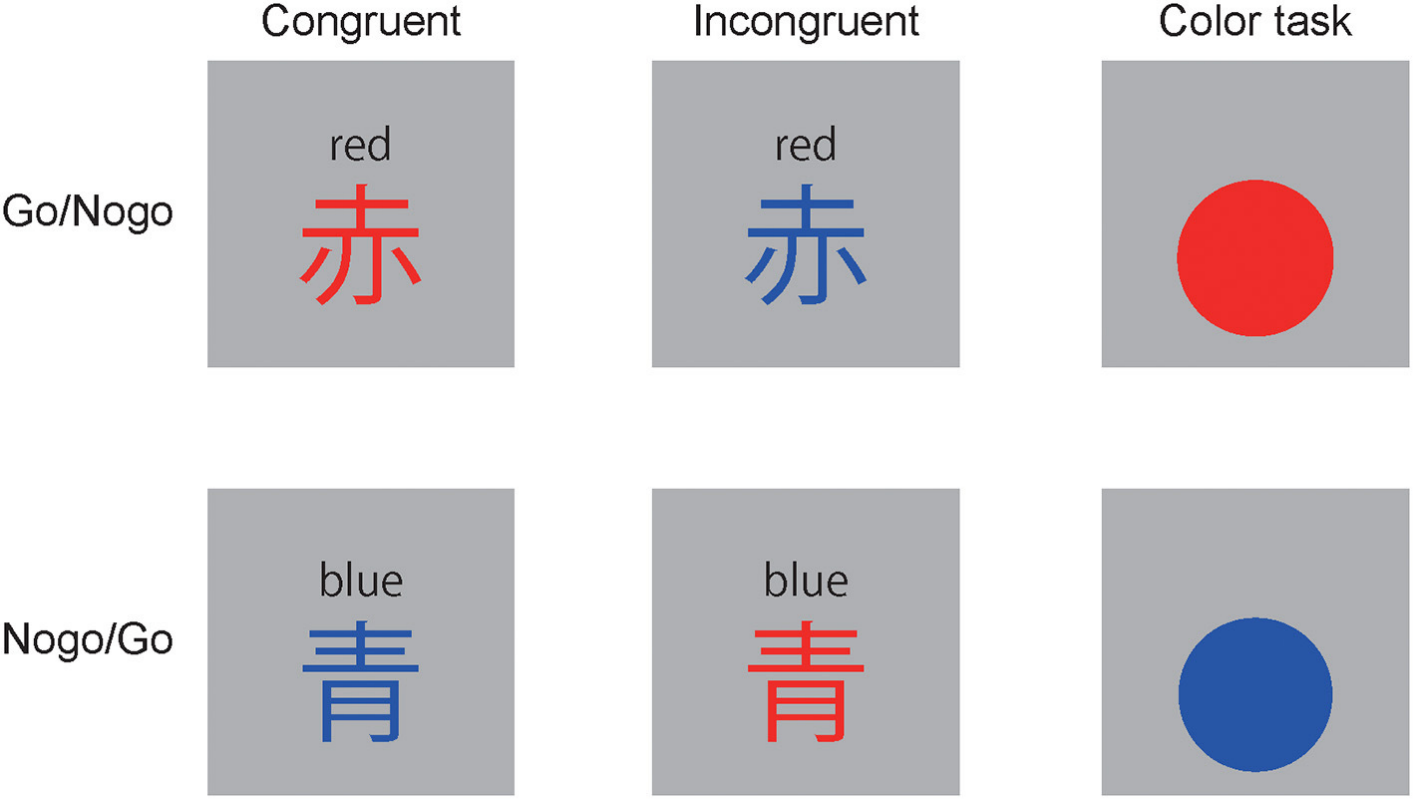
Tasks: 赤” means “red,” “青 means “blue.” Stimuli were presented for 100 ms with a stimulus onset asynchrony of 1,200-1,500 ms.

This study aimed to examine sequential congruency effects on ERP components for Go- and Nogo-stimuli. Therefore, the hybrid reverse Stroop Go/Nogo task was developed (Figure 1). We assumed that Nogo-N2 and Go-N2 are associated with cognitive control (Gajewski and Falkenstein, 2013; Huster et al., 2013; Suzuki et al., 2020), and intense conflicts require effortful cognitive control. Therefore, if N2 were independent of response inhibition task demands, then Nogo-N2 and Go-N2 would be modulated by sequential congruency effects. Nogo-P3 has been associated with response inhibition (Huster et al., 2013; Suzuki et al., 2020). We hypothesized that response inhibition is larger for incongruent stimuli than congruent ones, and it was enhanced for cI relative to iI. Nogo-P3 would change according to the modulation of the response inhibition effort. We also assumed that Go-P3 was enhanced for iC and cI than for cC and iI because Go-P3 was associated with contextual updating operations (Polich, 2007, 2012).

This study also examined the modulation of the N1 by the sequential congruency effect. A previous study of the flanker task demonstrated that the N1 was reduced by trials preceded by incongruent stimuli relative to congruent stimuli, which was explained as resulting from attentional control (Suzuki and Shinoda, 2015). Therefore, in the current experiment, it was assumed that the Go-N1 and the Nogo-N1 were modulated by the previous trial congruency.

## Method

### Participants

Undergraduate and graduate students (*N* = 21) participated in this study. They reported to have normal or corrected normal vision and normal color perception. Data of two participants were excluded from the analyses because their data had excessive electroencephalogram (EEG) artifacts. The data of 19 participants were used (sex: 10 males, 9 females; handedness: 16 right-handers, 3 left-handers). Their ages ranged from 18 to 23 years (mean ± SD, 19.63 ± 1.16 years). All participants gave their written informed consent before the experiment. The experimental procedure was approved by the ethics committee of Shitennoji University (2019-53).

### Task

The experiment was controlled using Psychtoolbox version 3 and GNU Octave. The stimuli in the hybrid reverse Stroop Go/Nogo task were two Kanji characters (width = 2.15, height = 2.16°), i.e., “赤” meaning “red,” and “青” meaning “blue.” The stimuli were painted in congruent or incongruent colors (Figure 1). Participants were asked to press a button using their thumb in the dominant hand in response to one of the two characters (i.e., Go-stimulus) and stop responding to the other character (i.e., Nogo-stimulus). The character, “赤” was the Go-stimuli for 10 participants, whereas “青” was the Go-stimuli for the others. The conditions of the Go/Nogo and the present trial congruency were set to equal frequencies. The trials were classified into eight conditions based on the Go/Nogo, the present trial congruency, and the previous trial congruency. The portion of trials ranged from 12.22 to 13.02% (Go cC: 12.70%, iC: 12.70%, cC: 12.70%, cC: 12.70%; Nogo cC: 12.86%, iC: 12.38%, iI: 13.02%, cI: 12.22%).

Color tasks were conducted before the reverse Stroop task block to increase attendance to colors and to enhance the reverse Stroop interference effect. Stimuli were circles (width= 2.15° and height = 2.15°) painted in red or blue (Figure 1). Participants were asked to press a button using their thumb in the dominant hand in response to one color and stop responding to the other color. Go- and Nogo-stimuli were equally presented.

The stimuli in both tasks were presented for 100 ms, and the stimulus onset asynchronies were randomly set between the range of 1,200 and 1,500 ms (step = 100 ms). A block consisted of 64 trials in the hybrid reverse Stroop Go/Nogo task, whereas it consisted of 16 trials in the color task. Participants performed 10 sets composed of one block of the color task and one block of the reverse Stroop task after conducting one set as a practice session.

### EEG Recordings and Analyses

Electroencephalograms were recorded from 31-channel Ag/AgCl electrode cap (i.e., Fp1, Fp2, F7, F3, Fz, F4, F8, T7, C3, Cz, C4, T8, FC3, FCz, FC4, P7, P3, Pz, P4, P8, PO8, PO3, POz, PO4, PO7, CP3, CPz, CP4, O1, Oz, and O2) using BIO-NVX 36 (Medical Computer Systems). The reference and ground channels were located at the nose tip and AFz, respectively. The sampling rate was 1,000 Hz, with impedances maintained under 10 kΩ.

Electroencephalograms and ERPs were analyzed using MATLAB R2019a (Mathworks inc.) and EEGLAB v2019.0 (Delorme and Makeig, 2004). Offline EEGs were re-referenced to the averages for all the channels and were bandpass filtered from 0.1 to 50 Hz (−6 dB/octave). Epochs were extracted from −100 to 1,000 ms triggered by stimulus onset, and means of durations from −100 to 0 ms were used as the baseline. Artifacts related to eye movements and muscle activities were excluded by pca and bsscca functions of the automatic artifact removal toolbox (Gómez-Herrero, 2007). Epochs with ± 50 μV were automatically excluded, and epochs contaminating artifacts (e.g., eye movements) were further excluded by a visual inspection. ERPs were computed for eight conditions (cC, iC, cI, and iI for Go-and Nogo-stimuli).

Temporal windows and channels of each ERP component were selected based on grand average ERP waveforms and topographical maps. Go-N1 and Nogo-N1 amplitudes were calculated as means from 150 to 200 ms at P7 and P8. Go-N2 and Nogo-N2 amplitudes were calculated as the means from 220 to 320 ms at Fz, FCz, and Cz. Go-P3 amplitudes were calculated as means of the two ranges from 300 to 400 ms and from 400 to 500 ms at Pz and POz, respectively. Nogo-P3 amplitudes were calculated as the means of the two ranges from 350 to 425 ms and from 425 to 500 ms at FCz, Cz, and Pz.

### Statistical Analyses

Repeated measures ANOVAs with the present trial congruency and the previous trial congruency were conducted on correct RT and commission error rates. Repeated measures ANOVAs, with channels, the present trial congruency, and the previous trial congruency, were conducted on Go-N1, Go-N2, Go-P3s, Nogo-N1, Nogo-N2, and Nogo-P3s amplitudes. Simple effect analyses were performed if the interaction between two variables was significant. Also, paired *t*-tests were used to examine differences in Nogo-N2 amplitudes between iC and cI and between iI and cC. Repeated measures ANOVAs were performed for the present trial congruency and the previous trial congruency in each channel if the interaction among the three variables were significant for ERP amplitudes. Bonferroni tests were used for *post-hoc* tests.

## Results

### Behavioral Results

**Figure 2:**
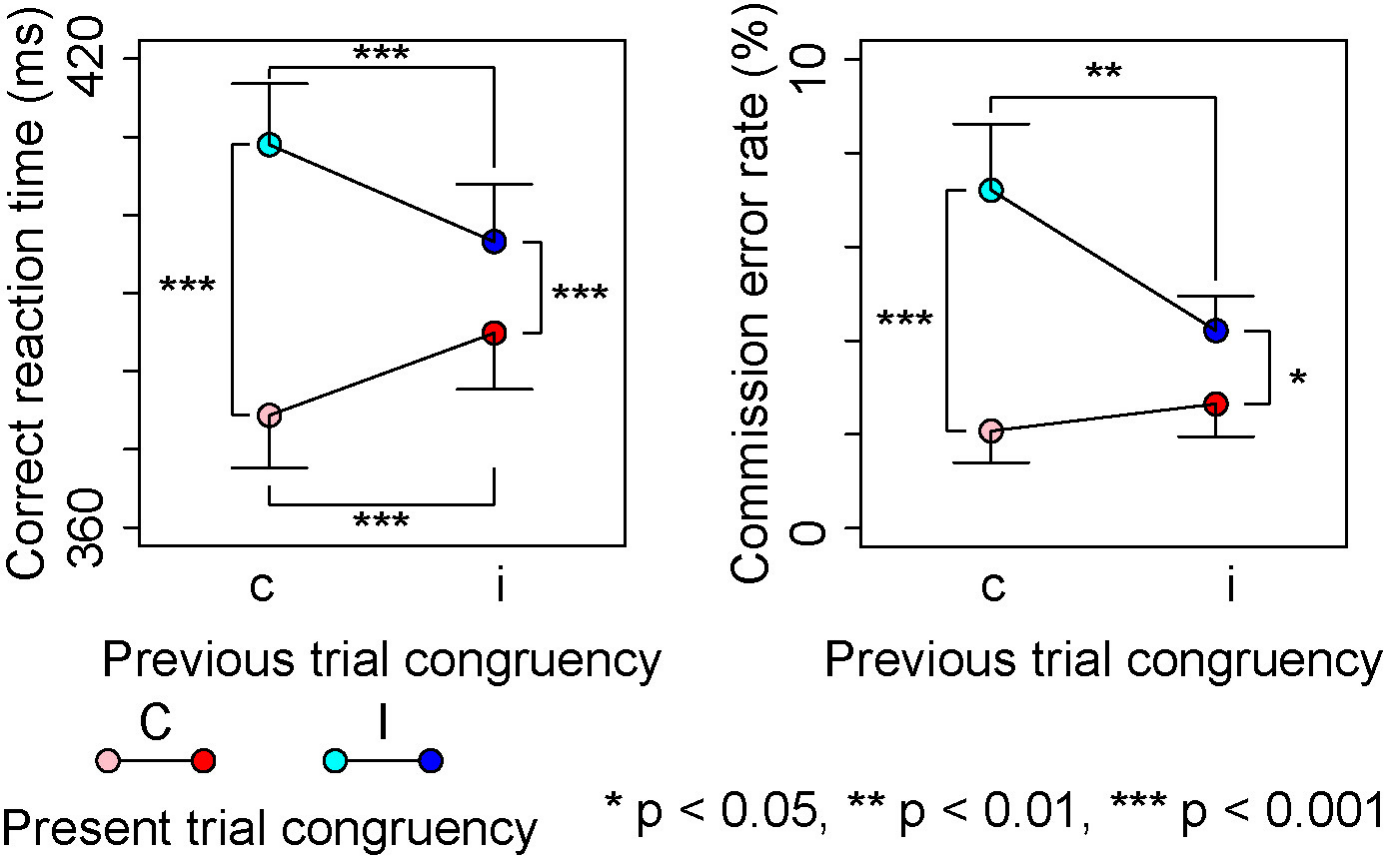
Behavioral results.

Figure 2 shows correct RTs for Go-stimuli and commission error rates for Nogo-stimuli in each condition. Omission error rates for Go-stimuli were nearly 0% in each condition (mean ± SD, cC: 0.20 ± 0.49%, iC: 0.34 ± 0.58%, cI: 0.47 ± 0.96%, iI: 0.20 ± 0.47%).

A repeated measures ANOVA on correct RTs showed a significant main effect of the present trial congruency [*F* (1,18) = 93.24, *p* < 0.001, $\eta_{p}^{2}$ = 0.84] and a significant interaction between the present and previous trial congruencies [*F* (1,18) = 71.88, *p* < 0.001, $\eta_{p}^{2}$ = 0.80]; however, there was no significant main effect of the previous trial congruency [*F* (1,18) = 0.37, *p* = 0.55, $\eta_{p}^{2}$ = 0.02]. A simple effect analysis showed a significant main effect of the present trial congruency on trials preceded by congruent stimuli [*F* (1,18) = 135.10, *p* < 0.001, $\eta_{p}^{2}$ = 0.88], on trials preceded by incongruent stimuli [*F* (1,18) = 21.72, *p* < 0.001, $\eta_{p}^{2}$ = 0.55], previous trial congruency on congruent stimuli in the present trial [*F* (1,18) = 34.15, *p* < 0.001, $\eta_{p}^{2}$ = 0.65], and incongruent stimuli in the present trial [*F* (1,18) = 29.73, *p* < 0.001, $\eta_{p}^{2}$ = 0.62]. These results indicated that the correct RT was the longest for cI, longer for iI than iC, and was the shortest for cC (Figure 2).

The repeated measures ANOVA on commission error rates showed a significant main effect of the present trial congruency [*F* (1,18) = 21.92, *p* < 0.001, $\eta_{p}^{2}$ = 0.55], and a significant interaction between present and previous trial congruencies [*F* (1,18) = 13.73, *p* < 0.01, $\eta_{p}^{2}$ = 0.43]; however, there was no significant main effect of the previous trial congruency [*F* (1,18) = 4.00, *p* = 0.06, $\eta_{p}^{2}$ = 0.18]. A simple effect analysis showed a significant main effects of the present trial congruency on trials preceded by congruent stimuli [*F* (1,18) = 23.43, *p* < 0.001, $\eta_{p}^{2}$ = 0.57], trials preceded by incongruent stimuli [*F* (1,18) = 6.73, *p* < 0.05, $\eta_{p}^{2}$ = 0.27], and the previous trial congruency on incongruent stimuli in the present trial [*F* (1,18) = 8.59, *p* < 0.01, $\eta_{p}^{2}$ = 0.32]; however, there was no significant main effect of the previous trial congruency on congruent stimuli in the present trial [*F* (1,18) = 2.23, *p* = 0.15, $\eta_{p}^{2}$ = 0.11]. These results indicated that commission error rates were the largest for cI and larger for cI than iC or CC (Figure 2).

### Go

**Figure 3:**
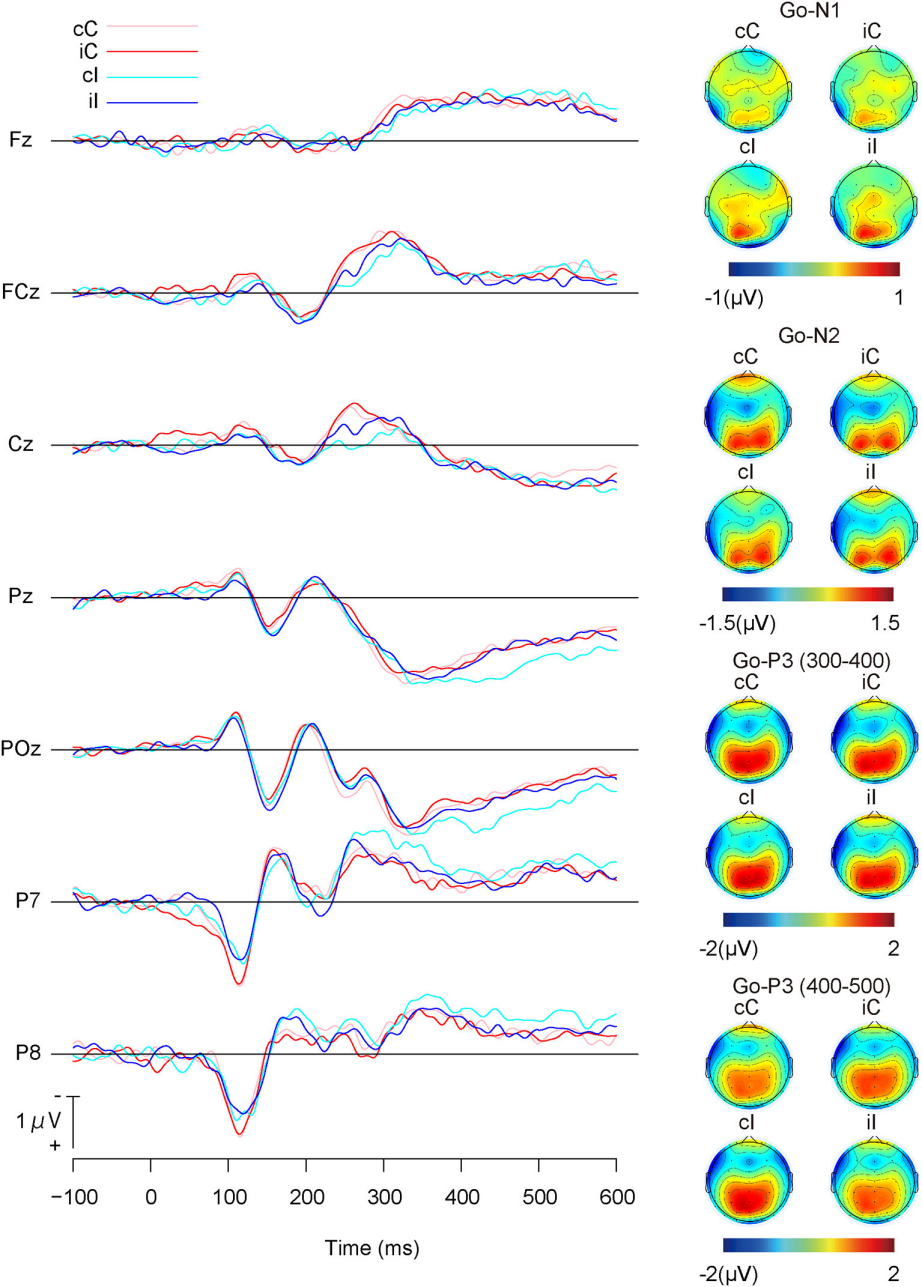
Grand average event-related potential (ERP) waveforms and topographies of Go-stimuli; cC: congruent (the previous trial) → Congruent (the present trial); iC: incongruent → Congruent; cI: congruent → Incongruent; and iI: incongruent → Incongruent.

Figure 3 shows the grand average ERP waveforms and the topographies of ERP components for Go stimuli, and Table 1 shows the means of the ERP amplitudes for Go stimuli. The repeated measures ANOVA on Go-N1 amplitudes indicated no significant main effects or interactions (Table 2).

**Table 1:** Means (SD) of event-related potential (ERP) amplitudes for Go-stimuli.

		**cC**	**iC**	**cI**	**iI**	**Main results (α = 0.05)**
Go-N1	P7	−0.74	−0.69	−0.50	−0.68	
(150–200 ms)		(1.80)	(1.55)	(1.73)	(1.82)	
	P8	−0.38	−0.29	−0.59	−0.55	
		(1.02)	(0.93)	(1.09)	(1.11)	
Go-N2	Fz	−0.17	−0.16	−0.07	−0.11	FCz > Fz at C
(220–320 ms)		(0.76)	(0.81)	(0.71)	(0.91)	C > I at FCz and Cz
	FCz	−0.75	−0.75	−0.36	−0.53	
		(0.86)	(0.90)	(0.98)	(0.97)	
	Cz	−0.47	−0.53	−0.07	−0.36	
		(0.85)	(0.89)	(1.10)	(0.82)	
Go-P3	Pz	1.48	1.41	1.53	1.43	
(300–400 ms)		(1.35)	(1.41)	(1.39)	(1.26)	
	POz	1.36	1.23	1.41	1.31	
		(0.88)	(0.82)	(0.84)	(0.91)	
Go-P3	Pz	0.97	1.06	1.51	1.06	cI > cC, iC and iI
(400–500 ms)		(1.23)	(1.32)	(1.40)	(1.25)	
	POz	0.87	0.90	1.38	0.98	
		(0.77)	(0.65)	(0.84)	(0.69)	

*cC: congruent (the previous trial) → Congruent (the present trial); iC: incongruent → Congruent; cI: congruent → Incongruent; and iI: incongruent → Incongruent*.

**Table 2:** The results of ANOVA of ERP components for Go-stimuli.

**Components**	**Variable**	**Statistical value**
Go-N1	A. Channels (P7,P8)	*F*(1,18) = 0.32, *p* = 0.58, *$\eta_{p}^{2}$* = 0.02
(150–200 ms)	B. Present trial congruency	*F*(1,18) = 0.34, *p* = 0.57, *$\eta_{p}^{2}$* = 0.02
	C. Previous trial congruency	*F*(1,18) = 0.00, *p* = 0.98, *$\eta_{p}^{2}$* = 0.00
	A×B	*F*(1,18) = 3.67, *p* = 0.07, *$\eta_{p}^{2}$* = 0.17
	A×C	*F*(1,18) = 0.83, *p* = 0.37, *$\eta_{p}^{2}$* = 0.04
	B×C	*F*(1,18) = 0.87, *p* = 0.36, *$\eta_{p}^{2}$* = 0.05
	A×B×C	*F*(1,18) = 0.51, *p* = 0.48, *$\eta_{p}^{2}$* = 0.03
Go-N2	A. Channels (Fz, FCz, Cz)	*F*(2,36) = 3.30, *p* < 0.05, *$\eta_{p}^{2}$* = 0.15
(220–320 ms)	B. Present trial congruency	*F*(1,18) = 5.69, *p* < 0.05, *$\eta_{p}^{2}$* = 0.24
	C. Previous trial congruency	*F*(1,18) = 0.89, *p* = 0.36, *$\eta_{p}^{2}$* = 0.05
	A×B	*F*(2,36) = 6.48, *p* < 0.01, *$\eta_{p}^{2}$* = 0.26
	A×C	*F*(2,36) = 2.39, *p* = 0.11, *$\eta_{p}^{2}$* = 0.12
	B×C	*F*(1,18) = 0.87, *p* = 0.36, *$\eta_{p}^{2}$* = 0.05
	A×B×C	*F*(2,36) = 0.92, *p* = 0.41, *$\eta_{p}^{2}$* = 0.05
Go-P3	A. Channels (Pz, POz)	*F*(1,18) = 0.36, *p* = 0.56, *$\eta_{p}^{2}$* = 0.02
(300–400 ms)	B. Present trial congruency	*F*(1,18) = 0.44, *p* = 0.52, *$\eta_{p}^{2}$* = 0.02
	C. Previous trial congruency	*F*(1,18) = 1.97, *p* = 0.18, *$\eta_{p}^{2}$* = 0.10
	A×B	*F*(1,18) = 0.17, *p* = 0.69, *$\eta_{p}^{2}$* = 0.01
	A×C	*F*(1,18) = 0.13, *p* = 0.72, *$\eta_{p}^{2}$* = 0.01
	B×C	*F*(1,18) = 0.00, *p* = 0.98, *$\eta_{p}^{2}$* = 0.00
	A×B×C	*F*(1,18) = 0.23, *p* = 0.64, *$\eta_{p}^{2}$* = 0.01
Go-P3	A. Channels (Pz, POz)	*F*(1,18) = 0.46, *p* = 0.51, *$\eta_{p}^{2}$* = 0.02
(400–500 ms)	B. Present trial congruency	*F*(1,18) = 14.47, *p* < 0.01, *$\eta_{p}^{2}$* = 0.45
	C. Previous trial congruency	*F*(1,18) = 3.98, *p* = 0.06, *$\eta_{p}^{2}$* = 0.18
	A×B	*F*(1,18) = 0.08, *p* = 0.77, *$\eta_{p}^{2}$* = 0.00
	A×C	*F*(1,18) = 0.00, *p = 0.9*9, *$\eta_{p}^{2}$* = 0.00
	B×C	*F*(1,18) = 9.85, *p* < 0.01, *$\eta_{p}^{2}$* = 0.35
	A×B×C	*F*(1,18) = 0.46, *p* = 0.51, *$\eta_{p}^{2}$* = 0.02

The repeated measures ANOVA on Go-N2 amplitudes revealed a significant interaction between channels and the present trial congruency (Table 2). A simple effect analysis showed a significant effect of channels on congruent stimuli in the present trial [*F* (2,36) = 5.27, *p* < 0.01, $\eta_{p}^{2}$ = 0.23], where Go-N2 amplitudes were significantly larger at FCz than Fz (*p* < 0.05). In addition, there were significant effects of the present trial congruency at FCz [*F* (1,18) = 7.18, *p* < 0.05, $\eta_{p}^{2}$ = 0.28] and Cz [*F*(1,18) = 7.04, *p* < 0.05, $\eta_{p}^{2}$ = 0.28], which indicated that Go-N2 amplitudes were significantly larger for congruent stimuli than for incongruent ones in the present trial at FCz and Cz. There were neither a significant effects of channels on incongruent stimuli in the present trial [*F* (1,18) = 1.75, *p* = 0.19, $\eta_{p}^{2}$ = 0.09] nor a significant effect of the present trial congruency on Fz [*F* (1,18) = 0.80, *p* = 0.38, $\eta_{p}^{2}$ = 0.04].

**Figure 4:**
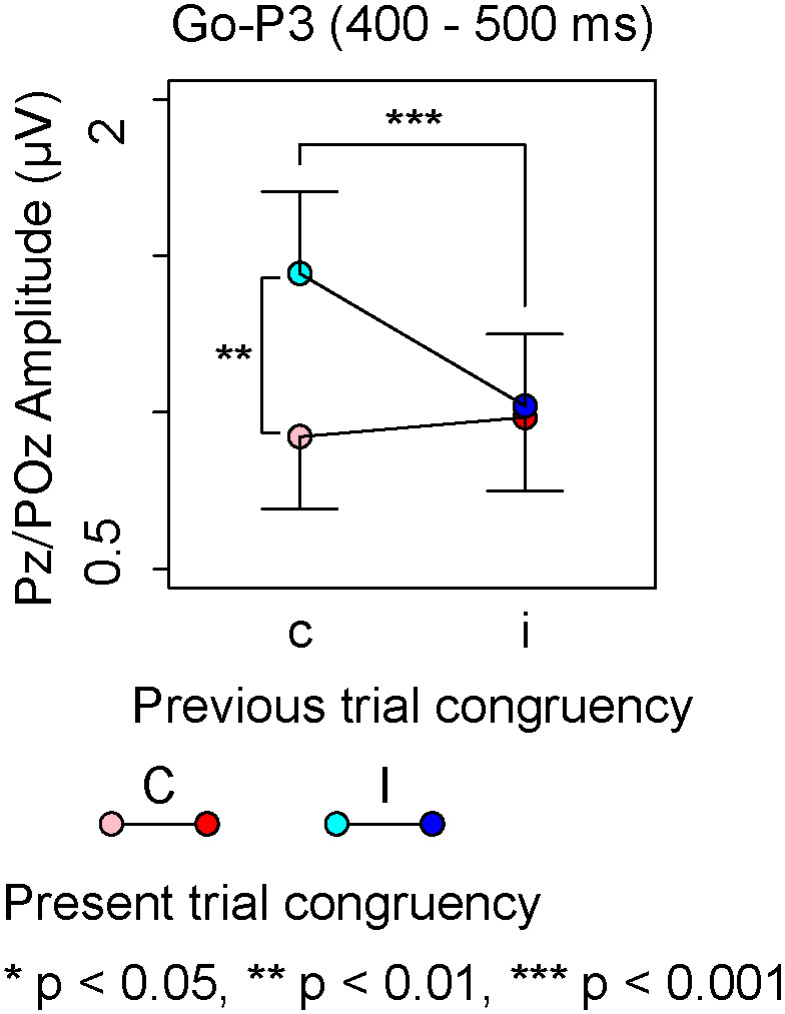
The main results of ERP components for Go-stimuli; “c” and “C” represent congruent stimuli on previous and present trials, respectively. “i” and “I” represent incongruent stimuli on previous and present trials, respectively.

There was neither a significant effect nor any significant interaction in the repeated measures ANOVA on Go-P3 amplitudes from 300 to 400 ms, whereas the repeated measures ANOVA on Go-P3 amplitudes from 400 to 500 ms indicated a significant interaction between present and previous trial congruencies (Table 2). A simple effect analysis showed a significant main effect of the present trial congruency on trials preceded by congruent stimuli [*F* (1,18) = 18.14, *p* < 0.001, $\eta_{p}^{2}$ = 0.50] and a significant main effect of the previous trial congruency on incongruent stimuli in the present trial [*F* (1,18) = 9.75, *p* < 0.01, $\eta_{p}^{2}$ = 0.35]. There were neither a significant main effect of the present trial congruency on trials preceded by incongruent stimuli [*F* (1,18) = 0.17, *p* = 0.68, $\eta_{p}^{2}$ = 0.01] nor a significant main effect of the previous trial congruency on incongruent stimuli in the present trial [*F* (1,18) = 0.38, *p* = 0.54, $\eta_{p}^{2}$ = 0.02]. These results indicated that Go-P3 amplitudes from 400 to 500 ms were the largest for cI (Figure 4).

### Nogo

**Figure 5:**
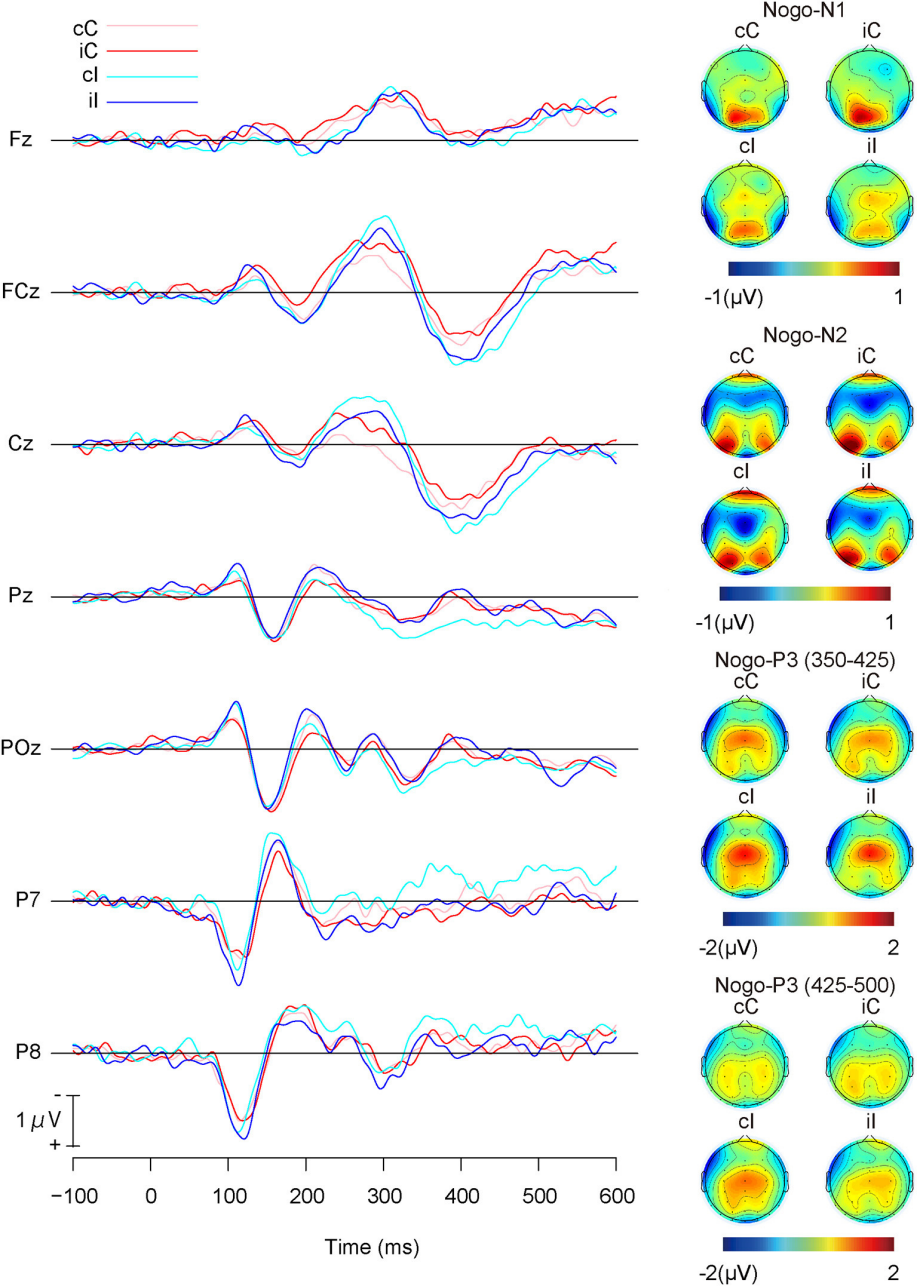
Grand average ERP waveforms and topographies of Go-stimuli; cC: congruent (the previous trial) → Congruent (the present trial); iC: incongruent → Congruent; cI: congruent → Incongruent; iI: incongruent → Incongruent.

Figure 5 shows the grand average ERP waveforms and the topographies of ERP components for Nogo-stimuli, and Table 3 shows the means of each ERP component for Nogo-stimuli. The repeated measures ANOVA on the Nogo-N1 amplitudes revealed a significant main effect of the previous trial congruency (Table 4), which indicated that Nogo-N1 amplitudes were significantly smaller in trials preceded by incongruent stimuli than congruent ones.

**Table 3:** Means (SD) of ERP amplitudes for Nogo-stimuli.

		**cC**	**iC**	**cI**	**iI**	**Main results (α = 0.05)**
Nogo-N1	P7	−0.81	−0.54	−0.99	−0.71	c > i
(150–200 ms)		(1.65)	(1.52)	(1.62)	(1.47)	
	P8	−0.56	−0.65	−0.72	−0.53	
		(0.96)	(0.92)	(0.99)	(1.12)	
Nogo-N2	Fz	−0.49	−0.61	−0.45	−0.38	FCz>Cz at C
(220–320 ms)		(0.63)	(0.71)	(0.81)	(0.82)	iC and cI>cC at FCz
	FCz	−0.49	−0.80	−0.90	−0.71	cI> iI and iC > cC at Cz
		(0.88)	(0.76)	(0.91)	(0.88)	
	Cz	0.03	−0.39	−0.76	−0.45	
		(1.08)	(0.90)	(0.66)	(0.86)	
Nogo-P3	FCz	0.76	0.61	1.07	1.07	FCz and Cz > Pz
(350–425 ms)		(0.95)	(0.91)	(1.01)	(1.13)	C > I
	Cz	1.09	0.93	1.42	1.34	cI > cC, cI > iI at Pz
		(1.09)	(1.15)	(1.38)	(1.29)	
	Pz	0.17	0.13	0.57	0.03	
		(0.81)	(0.75)	(0.76)	(0.65)	
Nogo-P3	FCz	0.11	0.08	0.70	0.36	I > C
(425–500 ms)		(0.54)	(0.68)	(0.82)	(0.69)	cI > iI > iC and cC
	Cz	0.42	0.45	1.04	0.71	
		(0.86)	(1.13)	(1.26)	(0.84)	
	Pz	0.20	0.24	0.53	0.20	
		(0.67)	(0.65)	(0.81)	(0.77)	

*Note. cC: congruent (the previous trial) → Congruent (the present trial); iC: incongruent → Congruent; cI: congruent → Incongruent; iI: incongruent → Incongruent*.

**Table 4:** The results of ANOVA of ERP components for Nogo-stimuli.

**Components**	**Variable**	**Statistical value**
Nogo-N1	A. Channels (P7,P8)	*F*(1,18) = 0.18, *p* = 0.68, *$\eta_{p}^{2}$* = 0.01
(150–200 ms)	B. Present trial congruency	*F*(1,18) = 0.48, *p* = 0.50, *$\eta_{p}^{2}$* = 0.03
	C. Previous trial congruency	*F*(1,18) = 11.45, *p* < 0.01, *$\eta_{p}^{2}$* = 0.39
	A×B	*F*(1,18) = 0.37, *p* = 0.55, *$\eta_{p}^{2}$* = 0.02
	A×C	*F*(1,18) = 2.60, *p* = 0.12, *$\eta_{p}^{2}$* = 0.13
	B×C	*F*(1,18) = 1.66, *p* = 0.21, *$\eta_{p}^{2}$* = 0.08
	A×B×C	*F*(1,18) = 1.15, *p* = 0.30, *$\eta_{p}^{2}$* = 0.06
Nogo-N2	A. Channels (Fz, FCz, Cz)	*F*(2,36) = 1.76, *p* = 0.19, *$\eta_{p}^{2}$* = 0.09
(220–320 ms)	B. Present trial congruency	*F*(1,18) = 2.59, *p* = 0.12, *$\eta_{p}^{2}$* = 0.12
	C. Previous trial congruency	*F*(1,18) = 0.47, *p* = 0.50, *$\eta_{p}^{2}$* = 0.03
	A×B	*F*(2,36) = 14.22, *p* < 0.001, *$\eta_{p}^{2}$* = 0.44
	A×C	*F*(2,36) = 0.13, *p* = 0.88, *$\eta_{p}^{2}$* = 0.01
	B×C	*F*(1,18) = 17.36, *p* < 0.001, *$\eta_{p}^{2}$* = 0.49
	A×B×C	*F*(2,36) = 7.69, *p* < 0.01, *$\eta_{p}^{2}$* = 0.30
Nogo-P3	A. Channels (FCz, Cz)	*F*(2,36) = 7.62, *p* < 0.01, *$\eta_{p}^{2}$* = 0.30
(350–425 ms)	B. Present trial congruency	*F*(1,18) = 20.06, *p* < 0.001, *$\eta_{p}^{2}$* = 0.53
	C. Previous trial congruency	*F*(1,18) = 12.15, *p* < 0.01, *$\eta_{p}^{2}$* = 0.40
	A×B	*F*(2,36) = 3.25, *p* = 0.05, *$\eta_{p}^{2}$* = 0.15
	A×C	*F*(2,36) = 2.74, *p* = 0.08, *$\eta_{p}^{2}$* = 0.13
	B×C	*F*(1,18) = 0.32, *p* = 0.58, *$\eta_{p}^{2}$* = 0.02
	A×B×C	*F*(2,36) = 4.18, *p* < 0.05, *$\eta_{p}^{2}$* = 0.19
Nogo-P3	A. Channels (FCz, Cz)	*F*(2,36) = 2.31, *p* = 0.11, *$\eta_{p}^{2}$* = 0.11
(425–500 ms)	B. Present trial congruency	*F*(1,18) = 21.19, *p* < 0.001, *$\eta_{p}^{2}$* = 0.54
	C. Previous trial congruency	*F*(1,18) = 5.54, *p* < 0.05, *$\eta_{p}^{2}$* = 0.24
	A×B	*F*(2,36) = 2.50, *p* = 0.10, *$\eta_{p}^{2}$* = 0.12
	A×C	*F*(2,36) = 0.09, *p* = 0.90, *$\eta_{p}^{2}$* = 0.01
	B×C	*F*(1,18) = 7.14, *p* < 0.05, *$\eta_{p}^{2}$* = 0.28
	A×B×C	*F*(2,36) = 0.02, *p* = 0.98, *$\eta_{p}^{2}$* = 0.00

**Figure 6:**
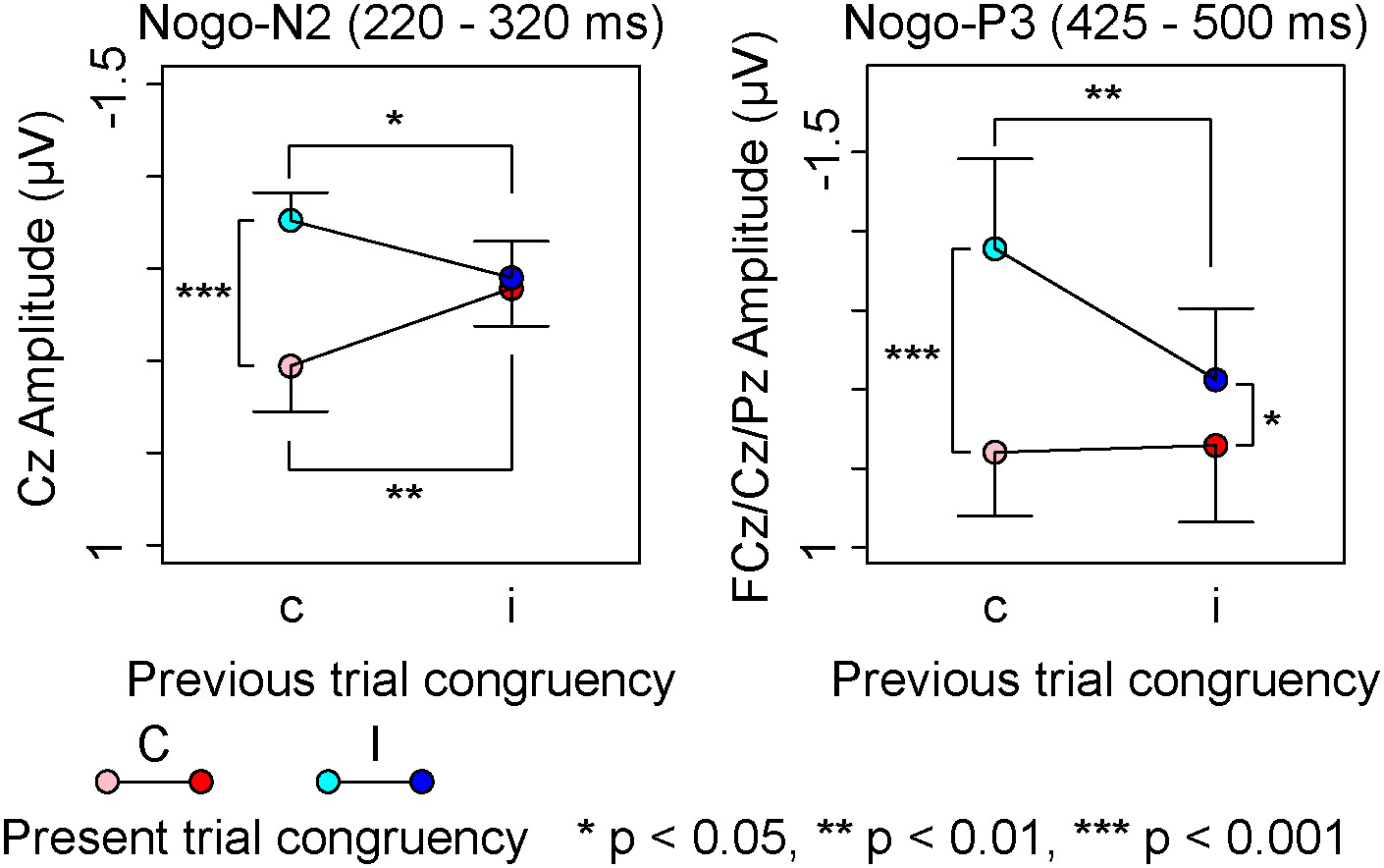
Main results of event-related potential components for Nogo-stimuli; “c” and “C” represents congruent stimuli on previous and present trials, respectively. “i” and “I” represents incongruent stimuli on previous and present trials, respectively.

A repeated measures ANOVA on Nogo-N2 amplitudes (Table 4) indicated a significant interaction among channels, the present trial congruency, and the previous trial congruency. The ANOVAs revealed a significant interaction between present and previous trial congruencies at FCz [*F* (1,18) = 11.36, *p* < 0.01, $\eta_{p}^{2}$ = 0.39] and Cz [*F* (1,18) = 26.62, *p* < 0.001, $\eta_{p}^{2}$ = 0.60], whereas the interaction was not significant at Fz [*F* (1,18) = 2.28, *p* = 0.15, $\eta_{p}^{2}$ = 0.11]. A simple effect analysis on Nogo-N2 amplitudes at FCz showed significant main effects of the present trial congruency on trials preceded by congruent stimuli [*F* (1,18) = 6.11, *p* < 0.05, $\eta_{p}^{2}$ = 0.25] and the previous trial congruency on congruent stimuli in the present trial [*F* (1,18) = 10.68, *p* < 0.01, $\eta_{p}^{2}$ = 0.37], whereas there were neither significant main effects of the present trial congruency on trials preceded by incongruent stimuli [*F* (1,18) = 1.08, *p* = 0.31, $\eta_{p}^{2}$ = 0.06] nor of the previous trial congruency on incongruent stimuli in the present trial [*F* (1,18) = 2.00, *p* = 0.17, $\eta_{p}^{2}$ = 0.10]. The simple effect analysis of Nogo-N2 amplitudes at Cz showed a significant main effect of the present trial congruency on trials preceded by congruent stimuli [*F* (1,18) = 28.16, *p* < 0.001, $\eta_{p}^{2}$ = 0.61], a significant main effect of the previous trial congruency on congruent stimuli [*F* (1,18) = 15.31, *p* < 0.01, $\eta_{p}^{2}$ = 0.46] and incongruent stimuli in the present trial [*F* (1,18) = 7.80, *p* < 0.05, $\eta_{p}^{2}$ = 0.30], whereas there was no significant main effect of the present trial congruency on trials preceded by incongruent stimuli [*F*(1,18) = 0.30, *p* = 0.59, $\eta_{p}^{2}$ = 0.02]. In addition, paired *t*-tests confirmed that N2 amplitudes at Cz were larger for iI than cC [*t* (1,18) = 3.29, *p* < 0.01] and cI than iC [*t* (1,18) = 2.91, *p* < 0.01]. These results indicated that Nogo-N2 amplitudes were the largest for cI, and the smallest for cC. Furthermore, there was no difference between Nogo-N2 amplitudes for iI and iC (Figure 6).

The repeated measures ANOVA on Nogo-P3 amplitudes from 350 to 425 showed significant main effects of channels and present and previous trial congruencies (Table 4). There was a significant interaction among channels, the present trial congruency, and the previous trial congruency. In addition, there was a significant interaction between present and previous trial congruencies at Pz [*F* (1,18) = 8.89, *p* < 0.01, $\eta_{p}^{2}$ = 0.33], whereas the interactions were not significant at FCz [*F* (1,18) = 0.49, *p* = 0.49, $\eta_{p}^{2}$ = 0.03] and Cz [*F* (1,18) = 0.13, *p* = 0.72, $\eta_{p}^{2}$ = 0.01]. The simple effect analysis of Nogo-P3 amplitudes from 350 to 425 at Pz showed significant main effects of the present trial congruency on trials preceded by congruent stimuli [*F* (1,18) = 11.86, *p* < 0.01, $\eta_{p}^{2}$ = 0.40] and the previous trial congruency on incongruent stimuli in the present trial [*F* (1,18) = 19.25, *p* < 0.001, $\eta_{p}^{2}$ = 0.52], whereas there was nether a significant main effect of the present trial congruency on trials preceded by incongruent stimuli [*F* (1,18) = 1.02, *p* = 0.33, $\eta_{p}^{2}$ = 0.05] nor previous trial congruency on congruent stimuli in the present trial [*F* (1,18) = 0.17, *p* = 0.69, $\eta_{p}^{2}$ = 0.01]. These results indicated that the Nogo-P3 amplitudes from 350 to 425 at Pz were larger for cI than cC or iI.

A repeated measures ANOVA on Nogo-P3 amplitudes from 425 to 500 showed significant main effects of present and previous trial congruencies and the interaction between them. Simple effect analysis indicated significant main effects of the present trial congruency on trials preceded by congruent stimuli [*F* (1,18) = 18.82, *p* < 0.001, $\eta_{p}^{2}$ = 0.51], trials preceded by incongruent stimuli [*F* (1,18) = 5.14, *p* < 0.05, $\eta_{p}^{2}$ = 0.22], and the previous trial congruency on incongruent stimuli in the present trial [*F* (1,18) = 10.27, *p* < 0.01, $\eta_{p}^{2}$ = 0.36], whereas there were no significant main effects of the previous trial congruency on congruent stimuli in present trials [*F* (1,18) = 0.05, *p* = 0.83, $\eta_{p}^{2}$ = 0.00]. These results indicated that Nogo-P3 amplitudes from 425 to 500 were larger for cI than iI, cI than cC, and iI than iC (Figure 6).

## Discussion

This study examined sequential congruency effects of reverse Stroop interference on ERP components for Go- and Nogo-stimuli using the hybrid reverse Stroop Go/Nogo task. The results indicated that the correct RT was the longest for cI, longer for iI than iC, and the shortest for cC; and the commission error rate was higher for cI than iI, and for incongruent stimuli than congruent stimuli. These behavioral results confirmed sequential congruency effects in the hybrid reverse Stroop Go/Nogo task.

Nogo-N1 amplitudes were smaller in trials preceded by incongruent stimuli than congruent stimuli. A previous study using the flanker task also demonstrated identical results, suggesting the inhibition of processing for the surrounding irrelevant non-targets (Suzuki and Shinoda, 2015). In addition, N1 amplitudes were larger in fluent Kanji readers compared with naïve Kanji readers (Niermeyer et al., 2018). Enhancement of N1 has been reported for color-discrimination tasks compared with simple reaction tasks (Vogel and Luck, 2000; Hopf et al., 2002), suggesting that attending to Kanji characters and the color is associated with the N1. Therefore, the current results suggest that inhibition of color processing was associated with the reduction of the Nogo-N1.

Contrary to the Nogo-N1, the Go-N1 was not modulated by the previous trial congruency. N1 amplitudes were larger in the discrimination task than in a simple reaction task (Vogel and Luck, 2000), suggesting that task demands influenced N1. Omission error rates were approximately 0% for incongruent Go-stimuli. In other words, response to Go-stimuli was easy; therefore, Go-stimuli might not need attentional control. Hence, it is suggested that the lack of task demands in response inhibition might be associated with low attentional control needs, which might eliminate the modulation of the Go-N1.

The Nogo-N1 was modulated by the previous trial congruency, which was not the case in the modulation of the Go-N1. Color processing interferes with word processing in the reverse Stroop task, and as a result, attentional control engages after inputting a stimulus. Interference in the flanker task occurs through the incongruence between the central target and the surroundings non-targets. Therefore, processing surrounding non-target locations can be proactively inhibited before an input, which might influence the N1 amplitude (Suzuki and Shinoda, 2015). As a result, the modulation of the Go-N1 may be observed in flanker interference. It is expected that future studies would examine differences in effects on ERP components for Go- and Nogo-stimuli based on interference types.

The Nogo-N2 amplitudes at Cz were larger for cI than iI. The Nogo-N2 is associated with cognitive control preceding motor responses, specifically cognitive control of conflict (Donkers and Van Boxtel, 2004; Smith et al., 2010; Randall and Smith, 2011; Groom and Cragg, 2015). The Nogo-N2 was modulated by sequential congruency effects, consistent with previous studies on interference tasks (Jiang et al., 2013; Panadero et al., 2015; Larson et al., 2016); however, commission error rate results suggested more intense conflicts for iI than iC, whereas there was no difference in N2 between iI and iC, suggesting that N2 was not directly associated with the conflict degree.

Previous studies have suggested that subcomponents of N2 reflect mismatches with the mental template (Folstein and Van Petten, 2008). The modulation of the Nogo-N2 was possibly associated with a mismatch with the mental template based on the previous trial congruency; however, the mismatch occurred for iC and cI of both the Go- and the Nogo-stimuli without the modulation of the Go-N2. Therefore, it was considered that task demands of response inhibition were necessary to modulate the N2 by sequential congruency effects. This study also showed that Nogo-N2 amplitudes were larger for cI than iC, suggesting that a high degree of effort for cognitive control increased Nogo-N2 amplitudes. Therefore, the Nogo-N2 might be involved in cognitive control, even though it was not directly associated with the conflict degree.

The Go-N2 decreased for incongruent than congruent stimuli in the present trial. It has been reported that the Go-N2 and the Nogo-N2 decrease because of task complexity (Mussini et al., 2020). The N2b is a subcomponent of the N2 and reflects selection processing (Smid et al., 1999). The N2b amplitudes were larger for target stimuli than non-target stimuli and were enhanced by relevant color non-target stimuli compared with irrelevant ones (Smid et al., 1999). The cognitive control effort was possibly reduced by the lack of response inhibition task demands for Go-stimuli, suggesting that the Go-N2 was more strongly associated with selection processing (i.e., N2b) than cognitive control. These findings suggest that the incongruent stimuli blurred target-related features and decreased Go-N2 amplitudes.

Previous studies have suggested that N1 and the N2 are modulated by sequential congruency effects (Jiang et al., 2013; Panadero et al., 2015; Suzuki and Shinoda, 2015; Larson et al., 2016). The tasks used in previous studies required participants to make a choice response and prevent inappropriate hand responses in all the trials. On the other hand, the task demands of response inhibition were absent for Go-stimuli in the hybrid reverse Stroop Go/Nogo task. In the present study, Nogo-N1 and Nogo-N2 were modulated by sequential congruency effects, but not Go-N1 or Nogo-N1. These findings indicate that sequential congruency effects on N1 and N2 require response inhibition task demands. Therefore, we suggest that attentional and cognitive control reflected by N1 and N2 were recruited based on response inhibition (or response control).

Nogo-P3 amplitudes from 425 to 500 ms were the largest for cI and were larger for iI than cC or iC. It was suggested that the Nogo-P3 is related to response inhibition (Gajewski and Falkenstein, 2013; Huster et al., 2013; Suzuki et al., 2020). Previous studies have demonstrated that the Nogo-P3 is modulated by sequential congruency effects (Jiang et al., 2013; Panadero et al., 2015; Larson et al., 2016). It is assumed that incongruent stimuli resulted in stronger response execution than congruent stimuli, which increased for cI relative to iI. Thus, the response inhibition effort might be stronger for cI than iI. It is suggested that the results of Nogo-P3 of this study are associated with changes in the response inhibition effort due to the sequential congruency effect.

The largest Go-P3 amplitudes from 400 to 500 ms were observed for cI, whereas there were no differences in Go-P3 amplitudes for cC, iC, or cI. The Go-P3 is related to context updating operations and subsequent memory storage (Polich, 2007, 2012). The results of Nogo-N1 indicated that incongruent stimuli triggered attentional control on the next trial; therefore, the context change from congruent to incongruent stimuli is crucial. On the other hand, context changes from incongruent to congruent stimuli were considered less important because congruent stimuli need not engage attentional control. Therefore, the enhancement of the Go-P3 in cI might involve a change in attentional control in the next trial.

Although the present sample size was larger than the median of previous ERP studies, a previous study indicated that the sample size was insufficient (Clayson et al., 2019). Hence, the findings of the current study need to be confirmed by future studies using larger samples. Since this study focused on the degree of conflict modulated by sequential congruency effects, we presented Go- and Nogo-stimuli at an equal frequency; however, the effects of stimuli congruency conflicts might be different for rare ERP components of Nogo-stimuli than frequent ones. It is suggested that future studies explore this issue. These results were inconsistent with the results of the previous studies, in which the degree of conflict was manipulated by Go/Nogo task probability and cues. One previous study modulated both Go-N2 and Nogo-N2 by the degree of conflict (Donkers and Van Boxtel, 2004; Smith et al., 2010; Randall and Smith, 2011). Thus, the effects of conflict on ERP components for Go- and Nogo-stimuli might be different between interference tasks and others. It is crucial to explore these differences in future studies.

This study examined sequential congruency effects on ERP components for Go- and Nogo-stimuli, using the hybrid reverse Stroop Go/Nogo task. The sequential congruency effects were interpreted as attentional control inhibiting color processing in trials preceded by incongruent stimuli, which reduced efforts for cognitive control and response inhibition (Botvinick et al., 2001). The Nogo-N1 was reduced by trials preceded by incongruent stimuli relative to congruent ones, suggesting the inhibition of color processing by incongruent stimuli on previous trials. Nogo-N2 amplitudes were larger for cI than iI and iC than cC, which might be related to cognitive control. On the other hand, the Go-N1 was not modulated by the previous trial congruency, and the Go-N2 was reduced on trials preceded by incongruent stimuli relative to congruent ones. These findings suggest that response inhibition task demand was necessary for the modulation of the N1 and the N2 by sequential congruency effects; however, both Go-P3 and Nogo-P3 amplitudes were the largest for cI. Therefore, the time range of ERP components might be related to the susceptibility of an interaction effect between response inhibition task demand and sequential congruency effects.

## Data Availability Statement

The data that support the findings of this study are available on request from the author, and approval by the ethics committee of Shitennoji University. The data are not publicly available because no informed consent was given by the participants for open data sharing.

## Ethics Statement

The studies involving human participants were reviewed and approved by ethics committee of Shitennoji University. The participants provided their written informed consent to participate in this study.

## Author Contributions

The author confirms being the sole contributor of this work and has approved it for publication.

## Conflict of Interest

The author declares that the research was conducted in the absence of any commercial or financial relationships that could be construed as a potential conflict of interest.

## Publisher's Note

All claims expressed in this article are solely those of the authors and do not necessarily represent those of their affiliated organizations, or those of the publisher, the editors and the reviewers. Any product that may be evaluated in this article, or claim that may be made by its manufacturer, is not guaranteed or endorsed by the publisher.
